# Effect of water storage on ultimate tensile strength and mass changes of universal adhesives

**DOI:** 10.4317/jced.53048

**Published:** 2017-01-01

**Authors:** Nazanin Bahrololumi, Amirreza Beglou, Ahmad Najafi-Abrandabadi, Alireza Sadr, Seyedeh-Mahsa Sheikh-Al-Eslamian, Amir Ghasemi

**Affiliations:** 1 Graduate Student, Research Institute of Dental Sciences, Dental school, Shahid Beheshti University of Medical Sciences, Tehran, Iran; 2 Faculty Member, Department of Restorative Dentistry, Dental School, Shahid Beheshti university of Medical Sciences, Tehran, Iran; 3 Acting Associate Professor, University of Washington School of Dentistry, 1959 NE Pacific St, Seattle, WA 98195, United States; Adjunct Associate Professor, International Exchange Center, Tokyo Medical and Dental University, 1-5-45, Yushima, Bunkyo-ku, Tokyo 113-8549, Japan; 4 Assistant Professor, Department of Restorative Dentistry, Dental School, Shahid Beheshti university of Medical Sciences, Tehran, Iran; 5 Full Professor, Preventive Dentistry Research Center, Research Institute of Dental Sciences, Department of Restorative Dentistry, Dental School, Shahid Beheshti university of Medical Sciences, Tehran, Iran

## Abstract

**Background:**

The aim of the present study was to evaluate the influence of water storage on micro tensile strength (µTS) and mass changes (MC) of two universal adhesives.

**Material and Methods:**

10 disk-shaped specimens were prepared for each adhesive; Scotchbond Universal (SCU) All-Bond Universal (ABU) and Adper Single Bond 2 (SB2). At the baseline and after 1 day and 28 days of water storage, their mass were measured and compared to estimate water sorption and solubility. For µTS test, 20 dumbbell shaped specimens were also prepared for each adhesive in two subgroups of 1 day and 28 days water storage.

**Results:**

MC was significantly lower for SCU and ABU than SB2 (*P* < 0.05) at both time intervals. In all three adhesives, the MC was significantly lower at 28 days compared to that at 1 day (*P* < 0.05). Similarly, µTS was significantly higher for SCU and ABU than SB2 at both storage intervals (*P* < 0.05). After 28 days, µTS increased significantly for universal adhesives (*P* < 0.05).

**Conclusions:**

MC and µTS of adhesives were both material and time dependent when stored in water; both universal adhesives showed less water sorption and higher values of µTS than the control group.

** Key words:**Absorption, dental adhesives, dentin-bonding agents, solubility, tensile strength.

## Introduction

Simplification of the procedures has always been favorable among dentists. Multimode one-bottle universal adhesives have been recently introduced to the dental market in this regard (1). They can be used in either Self-Etch or Total-Etch, or selective enamel etch mode (2). Their potential ability to bond to different restorative materials including zirconia (3), metal (4) and silica-based ceramics (5) is another advantage of these adhesives.

In order to produce universal adhesives, some alterations have been made in their formulation. In this regard, universal adhesives have approximately every element used in previous generations of adhesives in more complicated formulas (2) and are essentially similar to the one-step self-etch adhesives. The task of simplification in one-step self-etch adhesives have been made possible through increasing the amounts of solvents and hydrophilic functional monomers (6). In fact, presence of organic solvents and acidic monomers in the formulation of these new universal adhesives raises concerns about their water sorption (7). Water sorption is one of the most important factors responsible for adhesive degradation (8). Therefore, doubts still remain about their bonding durability (7), structural stability (9), formulation stability (10) and mechanical properties over time (11).

As demonstrated in many previous studies, physical properties of the adhesive may greatly affect the dentin-resin bond strength (12). A variety of tests are available to assess the physical properties of the adhesives, among which tensile strength has been widely used in literature (11,12).

Considering the recent introduction of these universal adhesives, there is relatively limited information regarding their performance and efficacy (1,13). Since specifically there was inadequate information covering their water sorption, solubility and tensile strength, the purpose of the present study was to evaluate the effect of water storage on micro tensile strength and mass change of two universal adhesives in a period of 28 days. A simplified etch-and-rinse adhesive was also tested as control group.

## Material and Methods

The study has been approved by the ethics committee of shahid behest university of medical sciences.

-Materials used

Three commercially available dental adhesive resins were used in this study. As the control material, Adper Single Bond 2) SB2; 3M ESPE, St. Paul, MN, USA) was used. The following two universal adhesive systems were tested: Scotchbond Universal Ad-hesive (SCU;3M ESPE, St.Paul, MN, USA); and All Bond Universal (ABU; Bisco Inc., Shaumburg, IL, USA). The compositions of these three dental adhesive resins are shown in  Table 1. The protocol for this study was mainly adopted according to ISO 4049, except for the dimensions of the specimens and water storage time.

**Table 1:**
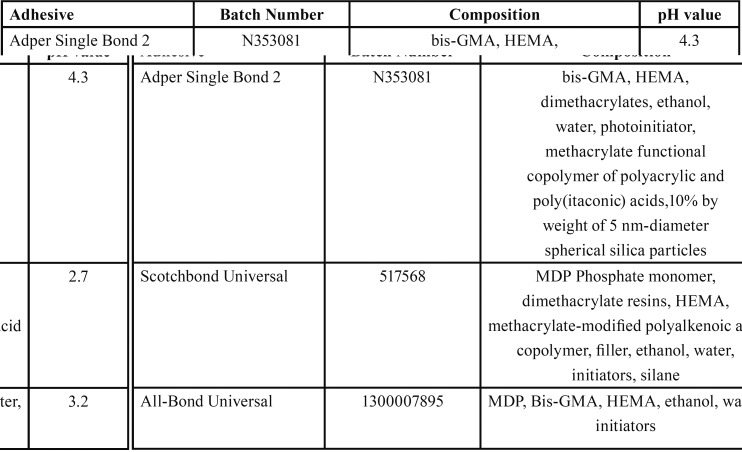
Adhesive system, batch number, composition and pH values of the adhesive systems according to the manufacturers’ material safety data issue or technical profile.

-Mass changes

▪Specimen preparation

Disk-shaped silicone molds (5.8mm diameter, 0.8mm thickness) were made by taking an impression from a plastic model. Thirty disks (10 of each adhesive) were prepared using these molds under standard laboratory conditions.

In order to produce the specimens of each adhesive, three drops of the adhesive were poured to fully fill up the mold in a dark and closed environment to avoid premature curing. Each drop was air dried for 40 seconds with oil/water free compressed air from a 10-cm distance, with 45° angle of the tip at 4 kg/cm2 output pressure to resemble the clinical situation (11). Care was taken to remove all visible bubbles. A plastic yarn (1 cm in length) was then placed into the resin to handle it in the upcoming stages. Then, to obtain a smooth surface, a glass slide was placed over the mold. The assembly was then sandwiched and clamped between two glass plates to exclude the atmospheric oxygen effect on polymerization.

The resin was first light-cured using a halogen light-curing unit (Optilux 501; Kerr, Danbury, CT, USA) at a power density of 650 mW/cm2 for 60 seconds, and then after removing the specimen from the mold, turned over and cured for further 60 seconds. The curing time was selected to ensure adequate light curing taking the bulk volume of resin into consideration.

After polymerization, the excess material around the disks was removed using a scalpel. Then, the specimens were observed under a stereo microscope (SMZ10; Nikon, Tokyo, Japan) at 16× magnification. Any specimen with visible voids or cracks was discarded. The resin disks were then stored in a desiccator at 37 ˚C for 24 hour. Each disk was repeatedly weighed in a digital scale (AL-104; Acculab, Mountville, PA, USA) with a reproducibility of 0.0001 gr until a stable mass (M0) was obtained. This was done as an assurance of complete vaporization of the solvent.

▪Measurement of mass changes (MC)

The specimens were soaked separately in sealed glass vials containing 10 ml of deionized water in an incubator (ON-300; AS-ONE, Tokyo, Japan) at 37˚C. After intervals of 24 hours and 28 days, the specimens were taken out using their plastic yarn, wiped with a soft absorbent paper, and then weighed immediately to record mass values after 1 day and 28 days of water storage (M1 and M28 respectively).

**Figure 1:**
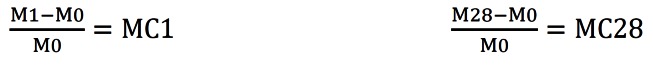
Formulae.

MC of the samples at each time point was calculated using the formulae below, (Fig. 1):

-Microtensile strength (µTS)

▪Specimen preparation

Silicone dumbbell-shaped molds (9 mm long, 3 mm wide and 0.65 mm thickness with isthmus wide of 0.8 mm) were made by taking an impression from a plastic model. Sixty specimens (20 of each adhesive) were prepared using these molds in ordinary laboratory environment.

Three drops of the adhesive were poured to completely fill the mold in a dark and closed environment in order to avoid premature curing. Care was taken to remove all visible bubbles. Active air-drying was conducted for all specimens to evaporate the solvent. Each drop was air dried for 40 seconds as represented for specimens in the previous section.

A glass slide was placed over the mold as described for specimens in the previous section, followed by a total of 120 seconds light curing, at 3 stages each covering 3×3 mm2 surface area of the specimen 40 seconds to ensure adequate light curing. After removing the excess material and discarding the specimens with visible voids or cracks, the specimen was stored in glass vials containing 10 ml of deionized water in two subgroups of 24 hours and 28 days (n=10).

▪Microtensile strength test

At the time of the test, the specimens were fixed with cyanoacrylate glue (Zapit; Dental Ventures of America, Corona, CA, USA) to a jig, and pulled apart in a micro tensile testing machine (Bisco micro tester; Bisco, USA) at a crosshead speed of 0.5 mm/min until fracture occurred.

The µTS was calculated in MPa, by dividing the load of failure (N) at the time of fracture by the cross-sectional area of the specimen at the fracture site (mm2).

▪Statistical analysis

The means and standard deviations of MC, and µTS were calculated for each dental adhesive. Kolmogorov-Smirnoff test was used to verify normality of the data. Repeated measures ANOVA was used to examine the effect of adhesive type and time of water storage on MC, and two-way ANOVA for the interaction of adhesives, time and µTS. Comparisons of each two groups were carried out using the Tukey’s test. Statistical significance for all tests was set at alpha = 0.05. Data analysis was carried out using SPSS software (version 16; SPSS, Chicago, IL, USA).

## Results

-Mass changes

Results are summarized in  Table 2 for the MC. All three adhesives showed the highest amounts of MC in the first day of water storage. For both 24 hours and 28 days of water storage, ABU showed the lowest MC values followed by SCU, and SB2 showed the highest levels of MC. On both time intervals, the differences in MC between SB2 and two other experimented adhesives were statically significant (*P*< 0.05), while there was no significant difference between the MC amounts of ABU and SCU (*P* = 0.610 and 0.116 for the 1st and 28th days of storage). On 28th day of storage all three adhesives showed a decrease in mass in comparison to the 1st day of storage, which was statically significant for all three adhesives (*P*< 0.05).

**Table 2:**
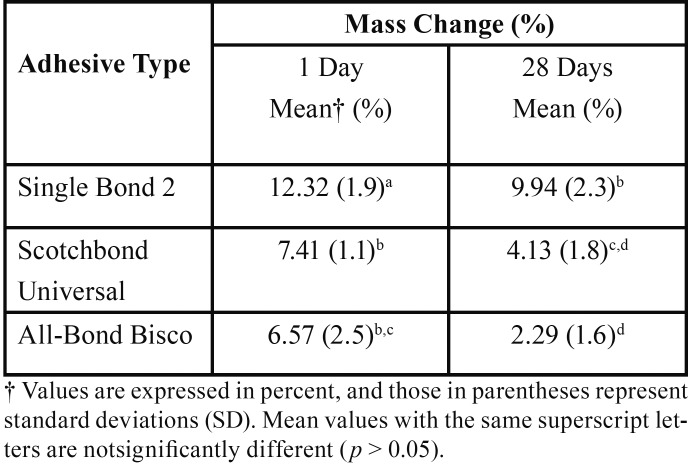
Mass change values (%) and standard deviations of experimented adhesives.

-Microtensile Strength

Results are summarized in  Table 3 for the µTS. SCU showed the highest amounts of µTS in both time intervals of water storage, followed by ABU and SB2 respectively. But, among these µTS values only the differences between that of SCU and ABU were not statistically significant (*P* = 0.130) and the differences between SB2 and ABU as well as SCU and SB2 (*P*< 0.05) were statistically significant. Also after 28 days of water storage the µTS values of SCU and ABU increased which were statically significant (*P* < 0.05).

**Table 3:**
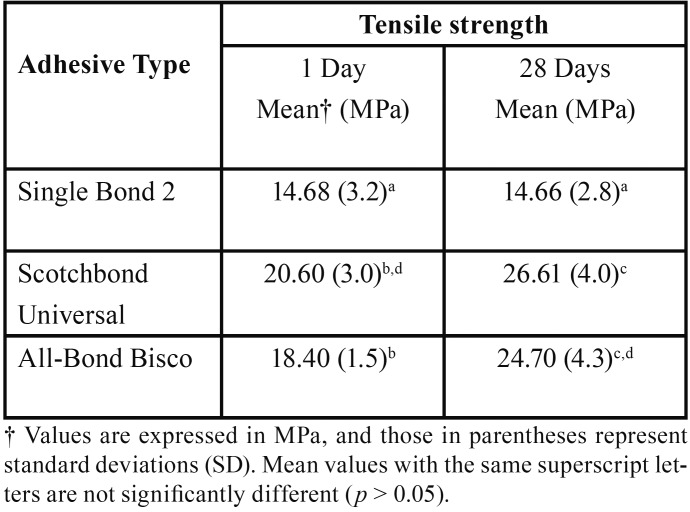
Micro Tensile Strength values and standard deviations of experimented adhesives.

## Discussion

The success of contemporary restorations is highly dependent on the properties of adhesive systems (14). For every new dental adhesive, the validity of its ideal claimed features should be verified. Therefore, the aim of this study was to evaluate the physical and mechanical properties of the relatively new universal adhesives. SCU and ABU were selected as widely available universal adhesives. Moreover, SB2 was selected among conventional two-step etch-and-rinse adhesives, because it has been considered as control group in other studies (13,15). There are some concerns about water sorption/solubility and mechanical properties of simplified adhesives such as universal adhesives that have all the components in a single bottle. Therefore, MC measurement and µTS test were selected to evaluate the physical and mechanical properties of these adhesives (11).

First and foremost, the present study showed that the three adhesives examined differed significantly in their MC and µTS when stored in water. Moreover, both MC and µTS values changed over time in all the three adhesives when stored in water.

All dental adhesives evaluated in this study absorbed a significant amount of water. The greatest increase in mass happened during the first day of storage, which is in agreement with previous studies (9,16,17). In both time intervals of 24 hours and 28 days, the highest values of MC were observed for SB2 followed by SCU and ABU respectively. Water sorption of dental adhesives is strongly influenced by resin composition and hydrophilicity (9,16,18,19). In fact, the chemistry of the monomers is the factor that determines the hydrophilic nature of a polymer (20). According to the product data sheets, there is an increased concentration of Bis-GMA in SCU compared to SB2 which explains lower water sorption of SCU and confirms the results obtained in this study. Bis-GMA is one of the most frequently used dimethacrylate monomers in adhesive systems which provides favorable features by forming densely cross-linked polymer. This monomer has both hydrophilic and hydrophobic components. Although some water sorption is inevitable because of its hydrophilic hydroxyl groups, Bis-GMA is mainly considered as a hydrophobic monomer which prevents substantial water uptake after water immersion (7). In addition to Bis-GMA, 10-MDP is another monomer widely employed in universal adhesives. This functional monomer is the most responsible monomer for chemical bonding to hydroxyapatite, and self-etching capability of the adhesive (13). Structurally, its long carbonyl chain makes this monomer relatively hydrophobic. As a consequence, water will be kept at a distance (21). On the hand, as shown in the product data sheets, SB2 has higher ethanol content. According to Malacarne *et al.* (17), addition of ethanol increased the ability of resin to absorb water. Although all three tested adhesives contain approximately the same concentration of HEMA (2-hydroxyethyl-methacrylate) which behaves as a hydrophilic monomer, the relevance of water sorption effect of this monomer in adhesive resin is controversial (7). We speculate that the universal adhesives SCU and ABU probably formed more hydrophobic networks, as they exhibited the lowest MC, which is in accordance with previous researches (9,17), claiming that the extent of water sorption decreases with the hydrophobicity of the resin blends.

On the other hand, absence of polyalkenoic acid copolymer in ABU and its lower content in SCU than SB2 is another reason suggesting the trend observed in our results for MC values. Polyalkenoic acid copolymer is a 3M ESPE adhesive component responsible for better moisture stability (22). However, it has previously been reported that this monomer does not have good solubility in adhesive solution and thus results in separate globules within the polymer preventing monomer approximation during polymerization and hence, causes water sorption of the adhesive due to decreased degree of conversion (11,13).

Moreover, in order to diminish the detrimental effect of acidic pH of self-etch adhesives on shelf life stability of the blend and enable room temperature storage of adhesives, most of the universal adhesives have a higher pH than traditional self-etch resins (10). SCU and ABU are considered as “mild self-etch” adhesives because of their relatively high pH (1). It is assumed therefore that universal adhesives are likely to have low polarity, which may lead to their relative low MC showed in this study.

While no statistically significant difference was observed between SCU and ABU in their MC values in the current investigation, the differences in their compositions seems to be the key reason for their slight difference in terms of MC. Munoz *et al*. (13,15) have demonstrated a lower degree of conversion for SCU, which tends to play an important role in elution of uncured monomer from the adhesive. Additionally, no amount of polyalkenoic acid copolymer and silane does exist in ABU composition. All these factors together with the higher pH of ABU are likely to result in lower MC of ABU in current study.

In contrast to some studies (7), after the period of 28 days, a decrease in MC values was observed for all the tested adhesives, with the same order of SB2 showing the highest value followed by SCU and ABU respectively. This finding is in accordance with the results of some other previous studies (9,17). Since the MC is the outcome of both the increase in mass caused by water sorption, and the decrease in mass caused by solubility, it is possible to conclude that the dissolution values over 28 days are greater than water uptake values. When stored in water, adhesive polymer absorbs water and results in network swelling (19,23). This event facilitates the leaching out of unreacted trapped monomers into the water (24). Furthermore, subsequent water sorption brings about polymer matrix degradation through the formation of nanopores (25). Elution of degradation products into surrounding water could be considered as solubility as well. For these reasons, It is suggestive that the release of monomers over time resulted in this decrease in mass (26,27). High amounts of HEMA in the formulation of adhesives, due to its low molecular weight and high solubility, may also associate in this phenomenon. The leakage of monomers into oral environment elicits concerns about the biocompatibility of these materials (28), and further research is needed in this regard.

It has been suggested that the degree of solvent evaporation may also affect the MC of adhesives; therefore additional sample were prepared in this study to examine the effect of prolonged solvent evaporation time on MC. Three additional disk-shaped resin specimens of each adhesive were prepared and left for 3 hours passively drying in a dark and closed place before light curing. These three samples of each adhesive exhibited lower water sorption after 24 hours compared to the active air dried ones. This decreased water sorption confirmed the effect of complete solvent evaporation on water stability of adhesives as proposed by some authors (11,16,23). It was suggested that the amount of remaining solvent affects greatly the chain topology and free volume spaces in the polymer and in turn, the water sorption of the polymer (7,9,23).The entrapment of remaining solvent has been thought to be responsible for producing localized areas of reduced degree of conversion, which is more prone to water absorption (16,23). It is noteworthy that in more hydrophilic resins the negative effects of residual ethanol on water sorption seemed to be greater, as shown by Malacarne *et al.* (17).

**Figure 2:**
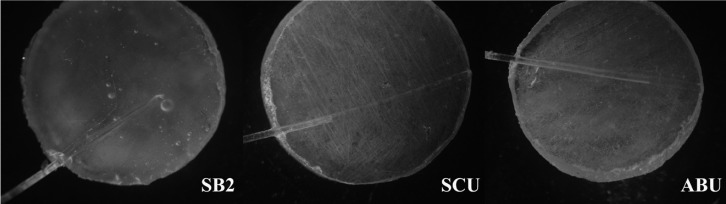
Stereomicroscope graphs of prepared specimens from three experimented adhesives.

One important observation worth mentioning is the stereomicroscope analysis of samples, which showed that more voids and cracks in SB2 disks were present after desiccation in comparison to the 2 universal adhesives (Fig. 2). As remained solvents evaporate during the desiccation cycle (27), the presence of solvent might result in voids in resin polymer seen by stereomicroscope (11). Possibly, this phenomenon may express the higher amounts of entrapped solvents after polymerization in the structure of SB2 disks.

Concerning with µTS of examined bonding adhesives, SCU showed the highest values of µTS as followed by ABU, and SB2 showed the lowest values in both time intervals of 24 hours and 28 days. In adhesives the resin matrix functions as a backbone providing mechanical properties. Apparently, the assumptions given above regarding the higher void formation, lower quality of polymer matrix and lower degree of conversion in SB2 should explain these results. Ye *et al.* (29) reported that with the increase of ethanol concentration in the adhesive formulation, the polymer crosslinking structure may change and tensile strength decrease which is again in line with our findings. According to the interaction theory of water sorption when a polymer is soaked in water, the bounded water to the resin structure is expected to cause a plasticizing effect which leads to the reduction of mechanical properties of the polymer (9,30). Probably due to these facts, in this study the material with the highest water uptake was also the one with the lowest µTS, which is in agreement with other studies (11,16). Interestingly, the µTS of the universal adhesives increased after 28 days of water storage which is in line with previous reports (11,23,29). It is suggested that the remaining free radicals continued to propagate and react the methacrylate double bonds after photo polymerization, which causes an increase in degree of conversion of the adhesive (11,29).
